# The influence of QTL allelic diversity on QTL detection in multi-parent populations: a simulation study in sugar beet

**DOI:** 10.1186/s12863-021-00960-9

**Published:** 2021-02-03

**Authors:** Vincent Garin, Valentin Wimmer, Dietrich Borchardt, Marcos Malosetti, Fred van Eeuwijk

**Affiliations:** 1 Biometris, Wageningen University and research Center, P.O Box 100, Wageningen, 6700AC The Netherlands; 2 KWS SAAT SE, Einbeck, Germany

**Keywords:** Multi-parent populations (MPPs), Quantitative trait locus (QTL), Allelic diversity, Simulation, R package mppR

## Abstract

**Background:**

Multi-parent populations (MPPs) are important resources for studying plant genetic architecture and detecting quantitative trait loci (QTLs). In MPPs, the QTL effects can show various levels of allelic diversity, which can be an important factor influencing the detection of QTLs. In MPPs, the allelic effects can be more or less specific. They can depend on an ancestor, a parent or the combination of parents in a cross. In this paper, we evaluated the effect of QTL allelic diversity on the QTL detection power in MPPs.

**Results:**

We simulated: a) cross-specific QTLs; b) parental and ancestral QTLs; and c) bi-allelic QTLs. Inspired by a real application in sugar beet, we tested different MPP designs (diallel, chessboard, factorial, and NAM) derived from five or nine parents to explore the ability to sample genetic diversity and detect QTLs. Using a fixed total population size, the QTL detection power was larger in MPPs with fewer but larger crosses derived from a reduced number of parents. The use of a larger set of parents was useful to detect rare alleles with a large phenotypic effect. The benefit of using a larger set of parents was however conditioned on an increase of the total population size. We also determined empirical confidence intervals for QTL location to compare the resolution of different designs. For QTLs representing 6% of the phenotypic variation, using 1600 *F*_2_ offspring individuals, we found average 95% confidence intervals over different designs of 49 and 25 cM for cross-specific and bi-allelic QTLs, respectively.

**Conclusions:**

MPPs derived from less parents with few but large crosses generally increased the QTL detection power. Using a larger set of parents to cover a wider genetic diversity can be useful to detect QTLs with a reduced minor allele frequency when the QTL effect is large and when the total population size is increased.

**Supplementary Information:**

The online version contains supplementary material available at (10.1186/s12863-021-00960-9).

## Background

The use of multi-parent populations (MPPs) for quantitative trait locus (QTL) detection is growing in popularity. With respect to bi-parental crosses, MPPs represent greater genetic diversity. With respect to association panels, the use of MPP designs increases the knowledge about the population structure. This information can be integrated in the QTL analysis to reduce the chance of false positive detection [1]. Here, we focus on MPPs composed of bi-parental crosses without further intercrossing. This definition does not cover MPPs like the multi-parent advanced generation inter-cross (MAGIC) populations [2]. Different statistical procedures exist to detect QTLs in MPPs but generally those methods, like the one adapting models used in genome-wide association studies [3, 4], do not model properly the diversity of allelic effects present in MPPs. Similarly, most of the MPPs simulation studies have not addressed the wide range of QTL allelic effects present in those populations. Therefore, we investigated the QTL detection power in MPPs using scenarios that accounted better for the MPP QTL allelic diversity.

### MPP design

**Fig. 1:**
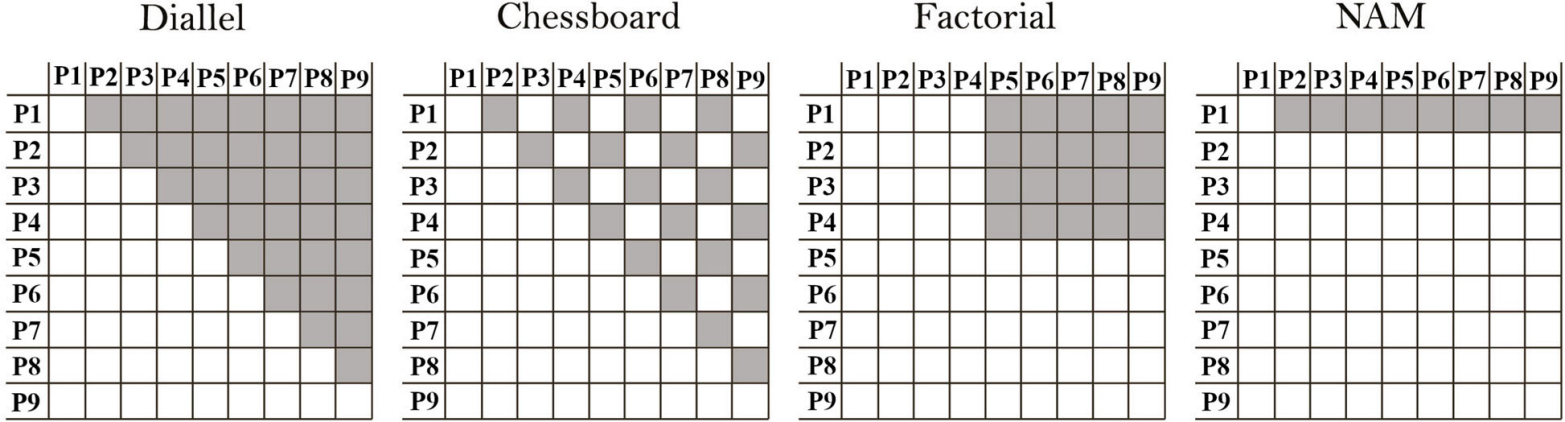
Diallel: regular pattern of pairwise crosses between a set of parents. Chessboard: diallel omitting a subset of crosses in diagonal fashion. Factorial: crosses between a set of parents A and another set of parents B. NAM: Crosses between a central parent and a set of peripheral parents

Many MPP designs have been evaluated through simulation studies [3, 5–7]. The nested association mapping (NAM) design is a collection of crosses between a central parent and peripheral lines [3] (Fig. 1). In a diallel design, each of *p* parent is crossed with the *p*−1 other parents [8]. In a factorial design, a set of parents A, is fully or partially crossed with another set of parents B [8].

To define an MPP design, we can look at the crossing scheme, the number of parents or crosses, and the number of individuals per cross. A larger set of parents covers a wider genetic diversity by sampling more alleles. For a given amount of resources (fixed total population size), more parents imply more crosses and therefore reduces the number of individuals per cross. When more alleles segregate, each allele has a lower frequency in the population. Therefore, the number of parents represents a trade-off between the number of alleles sampled and the sample size (total number of offspring lines in the breeding population) to detect their effect.

According to the literature, MPP designs with a reduced number of large crosses are more powerful [5, 9, 10]. Some authors tried to determine an optimal number of parents and of individuals per cross. For example, in diallel and single round robin designs, [11] found that the optimal number of parents followed from the crosses having 100 offspring lines. In MPPs with a fixed population size, [7] determined analytically that the detection power was only influenced by the number of parents and not by the MPP design. She showed however that the power reached a plateau after six parents. The trade-off between the required genetic diversity to be covered and the sample size to detect QTL alleles is a question that deserves further investigation. The answer to this question will be influenced by the QTL allelic diversity present in MPPs.

### QTL allelic diversity

**Fig. 2:**
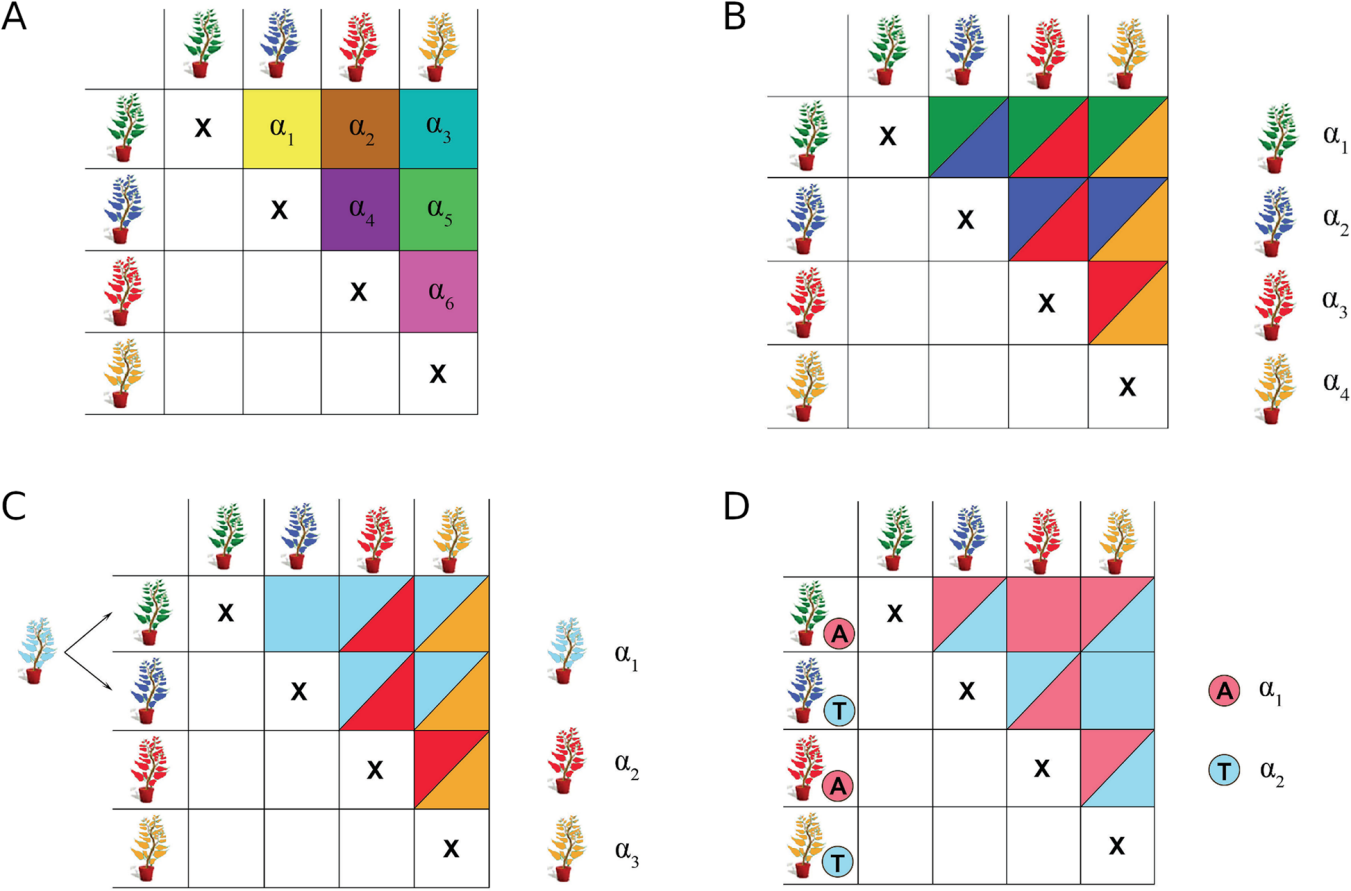
QTL effects illustration. **a**) cross-specific QTL: the allelic effects are different in every crosses due to some interaction between a QTL effect and the cross genetic background **b**) parental QTL: each parent carries a unique allele with consistent effect across the MPP **c**) ancestral QTL: parents inherit a reduced number of alleles due to shared ancestor. Each ancestral allele has a consistent effect across the MPP **d**) bi-allelic QTL: parent with the same SNP marker score are assumed to have inherited the same allele

In MPPs, since the crosses are derived from multiple parents, more alleles can potentially segregate with effects that are more or less diverse/consistent. We define four types of QTL allelic effects from the most diverse to the most consistent. In an MPP, the QTL effects can be defined in terms of allele origin and/or mode of action (Fig. 2). The first QTL allelic effect (cross-specific) represents an epistatic interaction between a QTL allele and a cross genetic background [12]. Thus, cross-specific QTL effects can only be estimated within crosses. The other QTL allelic effects are defined in terms of parental, ancestral, or single nucleotide polymorphism (SNP) alleles with consistent effects across crosses. The QTL allelic effects can be consistent because the alleles are specific to: 1) a common parent (parental), 2) a common ancestral line (ancestral), or 3) a common causal SNP (bi-allelic).

In the rest of the paper, we will refer to these four types of QTL effects calling them: cross-specific, parental, ancestral, and bi-allelic. Generally, the number of allelic effects that needs to be estimated decreases from the cross-specific to the bi-allelic QTLs. Therefore, the sample size required to accurately estimate the individual allelic or cross-specific QTL effects increases from the cross-specific to the bi-allelic QTLs. We hypothesize that the QTL allelic diversity has a strong influence on the detection of QTLs in MPPs.

### Statistical models

In MPPs, the choice of the statistical model used for QTL detection should consider the MPP design features and the variety of QTL effects. Xie et al. [10], Xu [9] and Verhoeven et al. [5] assumed cross-specific QTL effects, which statistically corresponds to a saturated model. Muranty [7] and Leroux et al. [13] considered the connection between crosses due to common parents using consistent parental QTL effects. Jansen et al. [6] and Klasen et al. [14] estimated multi-allelic QTL effects with an allele number between two and the number of parents. Liu et al. [11] used bi-allelic QTL models similar to the ones used in association studies. The Bayesian approach proposed by [15] is an elegant solution that estimates the number of alleles and the global QTL variance. However, according to the authors, with 600 individuals, it can only distinguish five alleles effectively. The computation in a Bayesian approach can also be intensive.

To capture the MPP QTL effect diversity, we used models assuming the cross-specific, parental, ancestral, and bi-allelic QTLs defined previously. We ran simulations based on genetic models with different levels of QTL allelic diversity that represent the genetic architectures in MPPs. We evaluated the performance of our models on four common MPP designs with five or nine parents to explore the ability to sample genetic diversity for QTL detection. Besides that, we also evaluated the effects of the population size, the QTL allelic diversity, the QTL effect size, and the detection model on the detection power, the resolution, and the false discovery rate. The results allowed us to provide guidelines to design MPPs in sugar beet. Those guidelines could be useful for other MPPs or crops.

## Methods

### MPP design

We evaluated the detection of QTLs on four MPP designs composed of *F*_2_ crosses: diallel, chessboard, factorial, and NAM (Fig. 1). Given a fixed total population size, these designs represent different strategies to sample the QTL allelic diversity. The diallel design maximizes the number of estimable allele genetic background interactions (each of *p* parent is used in *p*−1 crosses) but limits the number of individuals per cross. The chessboard design is a compromise that samples a reduced number of allele by background interactions (each parent is used in *p*/2 crosses if p is even, or [(*p*−1)/2] or [(*p*−1)/2]+1 crosses if p is odd) but allows more individuals per cross. The factorial design can be useful to cross two contrasting sets of parents (e.g. donors and recipients). Finally, the NAM design can be used to explore allelic diversity with respect to a reference parent [16]. In our case, the NAM design had the largest cross size.

We simulated the MPP designs using the genotypic data from nine parents coming from the sugar beet breeding program of KWS SAAT SE. Six parents were almost fully inbred with <1*%* of heterozygous markers and three were partially inbred with around 18% of heterozygous markers. The use of an existing set of parents provided a realistic basis to our simulations in terms of genetic properties. We simulated a reference diallel population composed of the 36 possible *F*_2_ crosses between the nine parents. Each cross contained 450 genotypes, with recombination and meiosis simulated using a random Poisson process based on the genetic map. For each *in silico* QTL mapping experiment, we simulated the QTL effects on the reference population and sampled the genotypes from that population to form realizations of the tested MPP designs. We fixed the total population size to 800 or 1600 and “crossed” either five or nine parents. The number of crosses varied from 4 to 36 and the cross size from 22 to 400 individuals (Table 1).

**Table 1:** MPP designs properties with the number of parents (N par), the number of crosses (N cr) and the number of individuals per crosses (N ind/cr)

MPP design	N par	N cr	N ind/cr (N = 800/1600)
Diallel	5	10	80/160
Diallel	9	36	22/44
Chessboard	5	6	133/266
Chessboard	9	20	40/80
Factorial	5	6	133/266
Factorial	9	20	40/80
NAM	5	4	200/400
NAM	9	8	100/200

### QTL genetic model

The QTL effects were simulated from a genetic map containing 5000 SNP markers spread across nine chromosomes for a total length of 968 cM (See Additional file 1). The average minor allele frequency (MAF) of the 9 parents SNP markers was 0.29 with values between 0.04 and 0.5. The average genetic distance between markers was equal to 0.2 cM with a maximum of 10.6 cM. We simulated seven types of QTLs (Table 2 and Additional file 2). Q1 and Q2 were cross-specific and had non-zero allelic effects in half and one third of the crosses, respectively. Setting some cross-specific effects to zero implies that the QTL does not interact with those backgrounds, which seems a reasonable assumption to make from our experience of analyzing real data. Q3 and Q4 were parental. Q3 had a different allelic effect for each parents, while Q4 only had a single non-zero parental allelic effect randomly assigned to one of the parents. Q5 and Q6 were ancestral, with ancestry groups determined by clustering the nine parental lines on local genetic similarity in a 10 cM window using the R package clusthaplo [13]. On average, we detected 3.9 ancestral alleles along the genome. While Q5 had a different allelic effect for each ancestral group, Q6 only had a single non-zero ancestral allelic effect randomly assigned. Q4 and Q6 were bi-allelic QTLs with a parental and ancestral basis. The last QTLs (Q7) were bi-allelic with an effect attached to the minor SNP allele.

**Table 2:** Simulated QTL effects described by type of allelic effect, segregation, number of alleles or QTL effects, and QTL genetic model

QTL	Allelic eff.	Segregation	N all. (Q. eff.)	Gen. mod.
Q1	cr. sp.	1/2 of the cr.	18	M1 (M5)
Q2	cr. sp.	1/3 of the cr.	12	M1 (M5)
Q3	parental	all parents	9	M2 (M5)
Q4	parental	1 parent	2	M2 (M5)
Q5	ancestral	all ancestors	4	M3 (M5)
Q6	ancestral	1 ancestor	2	M3 (M5)
Q7	bi-allelic	minor SNP allele	2	M4 (M5)

We sampled the non-zero QTL allelic effects from a uniform distribution (min=1; max=10) with the signs randomly assigned with an equal probability. In each *in silico* QTL mapping experiment, we simulated genetic models with eight QTLs. Each QTL was located on a different chromosomes keeping one chromosome free to study the false discovery rate. We simulated four QTLs with a small effect and four with a big effect representing 2% and 6% of the phenotypic variation, respectively. The total genetic contribution was always equal to 32% of the phenotypic variance. The sampled QTL allelic values were scaled to make the realized QTL variances equal to 2 or 6%. The remaining phenotypic variation representing the environmental and plot error was drawn from a normal distribution with proper variance. For details about the phenotype simulation see Additional files 3 and 4.

We simulated five QTL genetic models (M1-M5). The first four models used only QTLs with a single type of allelic effect. M1 contained only cross-specific QTLs (Q1 and Q2), M2 only parental QTLs (Q3 and Q4), M3 only ancestral QTLs (Q5 and Q6), and M4 only bi-allelic QTLs (Q7). In the last multi-QTL effects (MQE) model (M5), a combination of all above defined QTL effects was used. M5 contained one copy of Q1 to Q6 and two copies of Q7 to have two QTLs of each type. We assume that those models, especially the MQE model, will cover well the diversity of allelic effects in MPPs. In the rest of the paper, we will refer to the QTL genetic models calling them cross-specific, parental, ancestral, bi-allelic and MQE genetic models or we will refer directly to their types of QTL allelic effects.

### QTL detection model and procedure

The QTL detection models had the following form:

$$\boldsymbol{y} = \boldsymbol{X_{c}}\boldsymbol{\beta_{c}} + \boldsymbol{X_{Q}}\boldsymbol{\beta_{Q}} + \boldsymbol{r} $$

where ***X***_***c***_ represented a cross-specific intercept. ***X***_***Q***_ and ***β***_***Q***_ represented the QTL incidence matrix and the QTL effects that varied according to the number of QTL alleles or estimated effects [17]. We evaluated the QTL detection performances of four models assuming: cross-specific, parental, ancestral, and bi-allelic QTL allelic effects. The residual term ***r*** followed the linear model assumption of normality with a homogeneous variance $\boldsymbol {r} \sim N(0, \boldsymbol {I}\sigma _{r}^{2})$.

The QTL detection procedure was composed of a simple interval mapping scan to select cofactors. On each chromosome, we selected positions with −*log*10(*p*)>4. We applied an exclusion window of ±50 cM around the selected cofactors and iterated until no further QTLs were detected for inclusion in the cofactor set. The large exclusion window reduced the number of cofactors per chromosome to avoid model overfitting. Then, using the cofactors we performed a composite interval mapping to build a multi-QTL model. The QTLs were selected with an exclusion window of ±30 cM around the selected positions with the same procedure and threshold used for cofactors selection. The final list of QTLs was evaluated using a backward elimination.

The choice of a −*log*10(*p*)>4 threshold was not based on precise calculations but supported by values determined by permutation in real data analyses. For example, in [18], the threshold values varied between 3.4 to 5.6 for a type I error of 10%. The QTL detection scans were performed using the R package mppR [19].

### Evaluation statistics

We evaluated the QTL detection power at the whole genome level by calculating the true positive rate (TPR) as the number of simulated QTLs correctly detected divided by their total number (in %) (TPR = N simulated QTLs detected/N simulated QTLs). We assumed a detection window size around the simulated QTLs of ±5, 10, 20 cM, or the whole chromosome (TPR chr). We calculated the false discovery rate (FDR) at the whole genome level as the percentage of detected QTLs that were distant from a simulated QTL position by more than 5, 10 or 20 cM (FDR = N falsely detected QTLs/N detected QTLs). The false discovery rate on the chromosomes without simulated QTLs (FDR chr) was the percentage of runs where a QTL was wrongly detected on those chromosomes. The TPR denominator was the number of simulated QTLs (8) and the FDR denominator was the number of detected QTLs.

We evaluated the resolution of the QTL detection (dQTL) by measuring the distance between a simulated QTL and the largest significant peak on the chromosome. This information allowed us to determine a distribution for dQTL and to provide an ad hoc confidence intervals approximation for QTL positions detected in MPPs composed of *F*_2_ crosses. We defined the empirical confidence interval as *CI*_*α*_=2∗*dQTL*_*α*_, where *dQTL*_*α*_ is the *α* quantile of the dQTL distribution. The value *dQTL*_*α*_ is multiplied by two to account for situations where the detected QTL is positioned on the left or the right side of the true QTL. *CI*_*α*_ is the interval around the QTL position that contains the true QTL position with an *α* probability.

To evaluate the effect of the different simulation parameters, we computed analyses of variance (ANOVAs) with the TPR defined with a detection limit of at 10 cM as response and as explanatory factors: the MPP design (D), the number of parents (Np), the QTL detection model (M), the QTL size (Qs), and the type of QTL effect (Qe) (Model 1). We included in the model the two-way interactions between the MPP design, the number of parents, the QTL detection model, and the QTL size. The error term was assumed to be normally distributed $e \sim N(0, \boldsymbol {I}\sigma _{e}^{2})$.

1 $$\begin{array}{*{20}l}  TPR &= D + Np + M + Qs + Qe + D \times Np \\ &\quad+ D \times M + D \times Qs + Np \times M \\ &\quad+ Np \times Qs + M \times Qs + e  \end{array} $$

We interpreted the ANOVA results by calculating least-squares means, following a model selection procedure that retained significant interactions and significant main effects as well as non-significant main effects that underlie significant interactions. Least-squares means allowed us to get predictions averaged over a set of parameters to understand their effects on the TPR. Model 1 evaluates jointly the effect of all parameters and determines their relative contribution while, in most of the MPP simulations, the influence of each parameter was analyzed one by one. We computed the least-squares means using the R package emmeans [20].

We performed 50 replications of each *in silico* QTL mapping experiment for a different QTL genetic model (M1-M5). For each replication, we sampled MPPs given the population sizes (N = 800 or N = 1600), the MPP design (diallel, chessboard, factorial and NAM), and the number of parents (five or nine). On each sampled MPP, we performed QTL detection by the cross-specific, parental, ancestral, and bi-allelic models. It represented a total of 16,000 calculated QTL profiles with 128,000 simulated QTL positions. An R script reproducing all simulation steps is available in Additional file 4. Due to restriction about data availability we used the EU-NAM [21] parent genetic data for the companion example.

## Results

### Global measurements

Table 3 contains the average TPR and FDR at 5, 10, and 20 cM, and the average dQTL per population size and per QTL genetic model. The results are averaged over all other parameters (MPP design, number of parents, QTL detection model, QTL size, and QTL type). The TPR increased with the window size around the simulated QTL. However, the increase between the TPR at 20 cM and TPR chr (no minimum distance) was limited.

**Table 3:** Average TPR, FDR and dQTL results. The TPR and FDR are measured with detection window sizes around the simulated QTL of ±5, 10 and 20 cM, or the whole chromosome (TPR chr). FDR chr is the average FDR on the chromosome with no simulated QTL. The results are presented per population size (N = 800 and N = 1600) and QTL genetic model (cross-specific, parental, ancestral, bi-allelic, and MQE)

	N = 800					N = 1600				
Genetic model	Cr. sp.	Par.	Anc.	Biall.	MQE	Cr. sp.	Par.	Anc.	Biall.	MQE
TPR (*%*)(5*cM*)	24	16	25	26	23	42	34	48	50	42
FDR (*%*)(5*cM*)	45	47	35	32	40	42	41	26	24	33
TPR (*%*)(10*cM*)	32	22	30	31	29	52	43	56	56	51
FDR (*%*)(10*cM*)	28	28	21	19	24	28	24	14	13	20
TPR (*%*)(20*cM*)	38	27	35	35	34	61	51	61	61	58
FDR (*%*)(20*cM*)	12	11	8	7	10	13	10	6	5	9
TPR chr (*%*)	41	30	37	37	37	66	56	64	63	62
FDR chr (*%*)	0.3	0.9	0.2	0.6	0.5	1.7	0.9	0.1	0.6	0.7
dQTL (cM)	7.1	8	6.1	5.3	7.1	6.5	6.8	4.3	3.9	5.4

FDR chr (no simulated QTL) varied between 0.1 and 1.7*%*. On the chromosomes where QTLs were simulated, the FDRs were larger (e.g. at 10 cM around 25% and 20% for the N = 800 and N = 1600 populations respectively). The FDR decreased from the cross-specific and parental QTLs to the ancestral and bi-allelic ones. This tendency was more pronounced in the N = 1600 populations. For example, at 10 cM, the FDR was equal to 28%, 24%, 14% and 13% for the cross-specific, parental, ancestral, and bi-allelic genetic models, respectively.

dQTL followed a trend similar to the FDR and decreased from the cross-specific and parental QTLs to the ancestral and bi-allelic ones. For example, in the N = 1600 populations, dQTL decreased from 6.5 cM to 3.9 cM for the cross-specific and bi-allelic QTLs, respectively. In Table 4, we calculated the empirical confidence intervals for the 90, 95, and 99 percentile values of the dQTL distribution per QTL and population size for each genetic model. For an illustration of the dQTL distributions, see Additional file 5. Table 4 gives an estimation of the QTL confidence intervals. For example, in a population of N = 800, with a mix of QTL effects (MQE) explaining each 6% (2%) of the phenotypic variation, the 95% confidence interval was equal to 46 cM (70 cM). Finally, we noticed that increasing the population size from 800 to 1600 reduced dQTL more for the ancestral and bi-allelic QTLs than the cross-specific and parental ones. In general, the TPR, FDR, and dQTL results obtained for the MQE genetic model seemed to be intermediate to the other genetic models.

**Table 4:** Empirical confidence intervals in cM based on the 90, 95 and 99 percentile values from the dQTL distribution per QTL size (small 2% and big 6%), per QTL genetic model (cross-specific, parental, ancestral, bi-allelic, and MQE), and per population size (N = 800 and N = 1600)

		small QTLs (2%)					big QTLs (6%)				
		Cr. sp.	Par.	Anc.	Biall.	MQE	Cr. sp.	Par.	Anc.	Biall.	MQE
	.90	42	48	49	47	53	33	38	30	26	34
N=800	.95	60	61	65	65	70	49	53	41	39	46
	.99	114	109	107	141	110	83	105	78	64	81
	N det. QTL	1461	802	1030	954	1142	3768	3050	3734	3777	3627
	% det. QTL	23	13	16	15	18	59	48	58	59	57
	.90	36	43	34	32	37	34	32	17	15	25
N=1600	.95	54	58	49	47	51	49	46	26	25	39
	.99	95	93	91	81	88	85	79	56	49	69
	N det. QTL	3271	2130	2782	2624	2658	5113	5012	5365	5487	5217
	% det. QTL	51	33	43	41	42	80	78	84	86	82

### MPP design

**Fig. 3:**
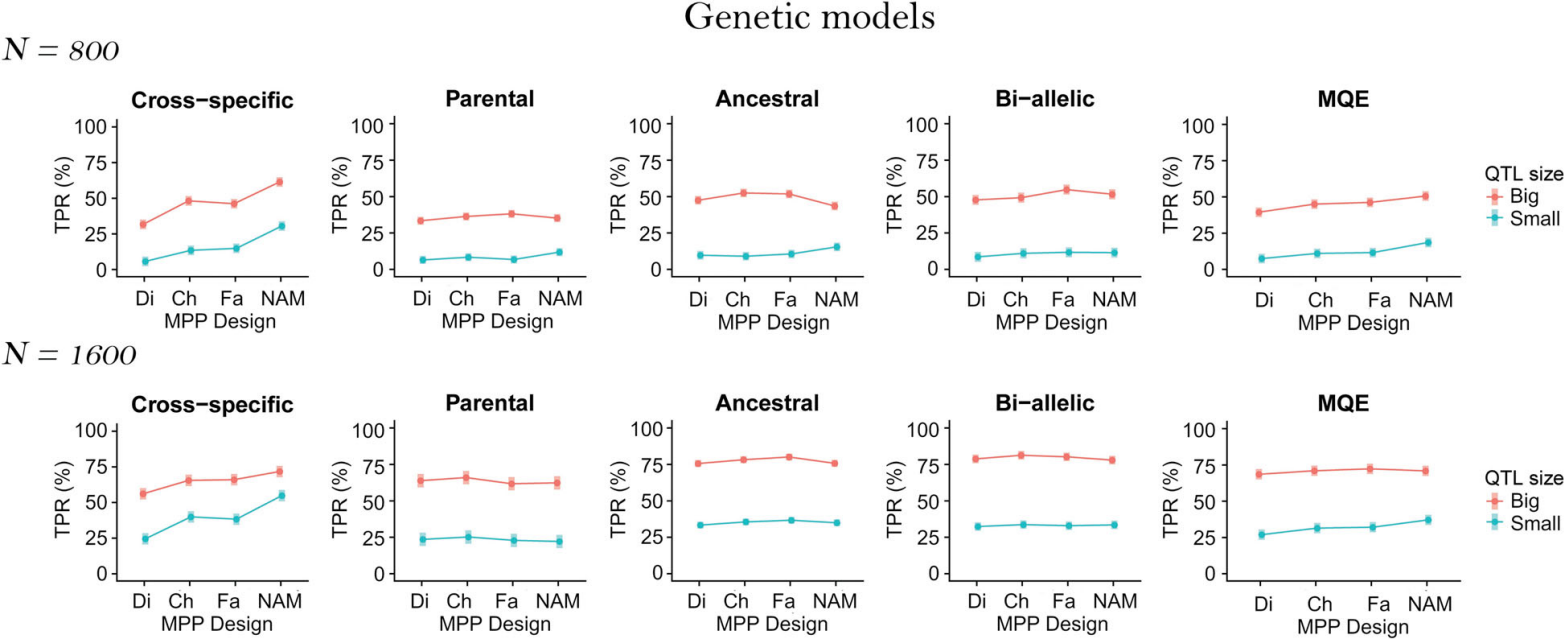
TPR least-squares means over the MPP designs (diallel, chessboard, factorial and NAM) and the QTL sizes (small 2% and big 6%) for all QTL genetic models (cross-specific, parental, ancestral, bi-allelic, and MQE) per population size (N = 800 and N = 1600)

In Fig. 3, we plotted the TPR least-squares means over the MPP design per QTL size for the N = 800 and N = 1600 populations using the ANOVA results from model 1. Detailed ANOVA results and F-statistics can be found in Additional file 6. The TPR least-squares means show that the MPP design was most influential for the cross-specific QTLs. The cross-specific QTLs are the only ones for which the TPR increases significantly from the diallel to the NAM design.

### Number of parents

**Fig. 4:**
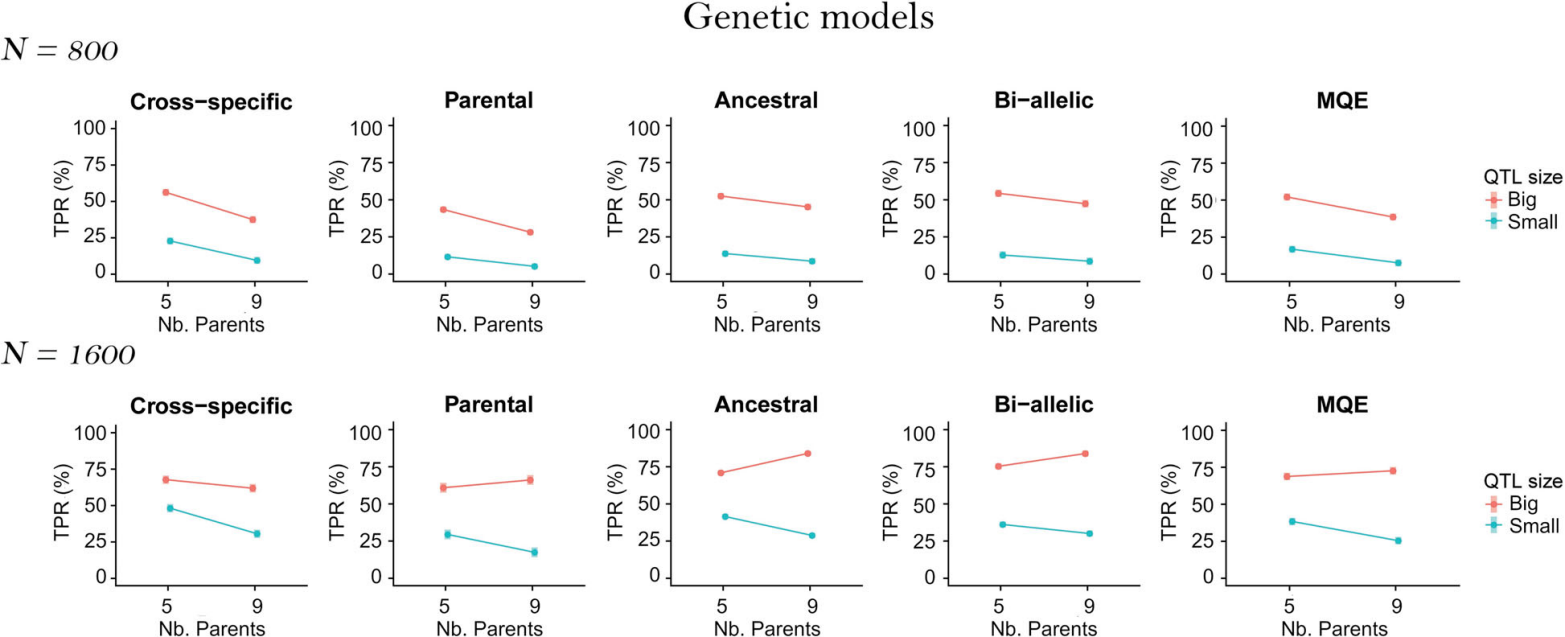
TPR least-squares means over the number of parents (5 and 9) and the QTL sizes (small 2% and big 6%) for all QTL genetic models (cross-specific, parental, ancestral, bi-allelic, and MQE) per population size (N = 800 and N = 1600)

To evaluate the effect of the parent numbers, we plotted the TPR least-squares means from model 1 against the number of parents and the QTL size in Fig. 4. We could observe that generally the MPP designs with nine parents had a lower TPR. This trend was consistent in the N = 800 populations. However, in the N = 1600 populations, for the big QTLs, the TPR increased from five to nine parents for the parental, ancestral, and bi-allelic QTLs.

**Fig. 5:**
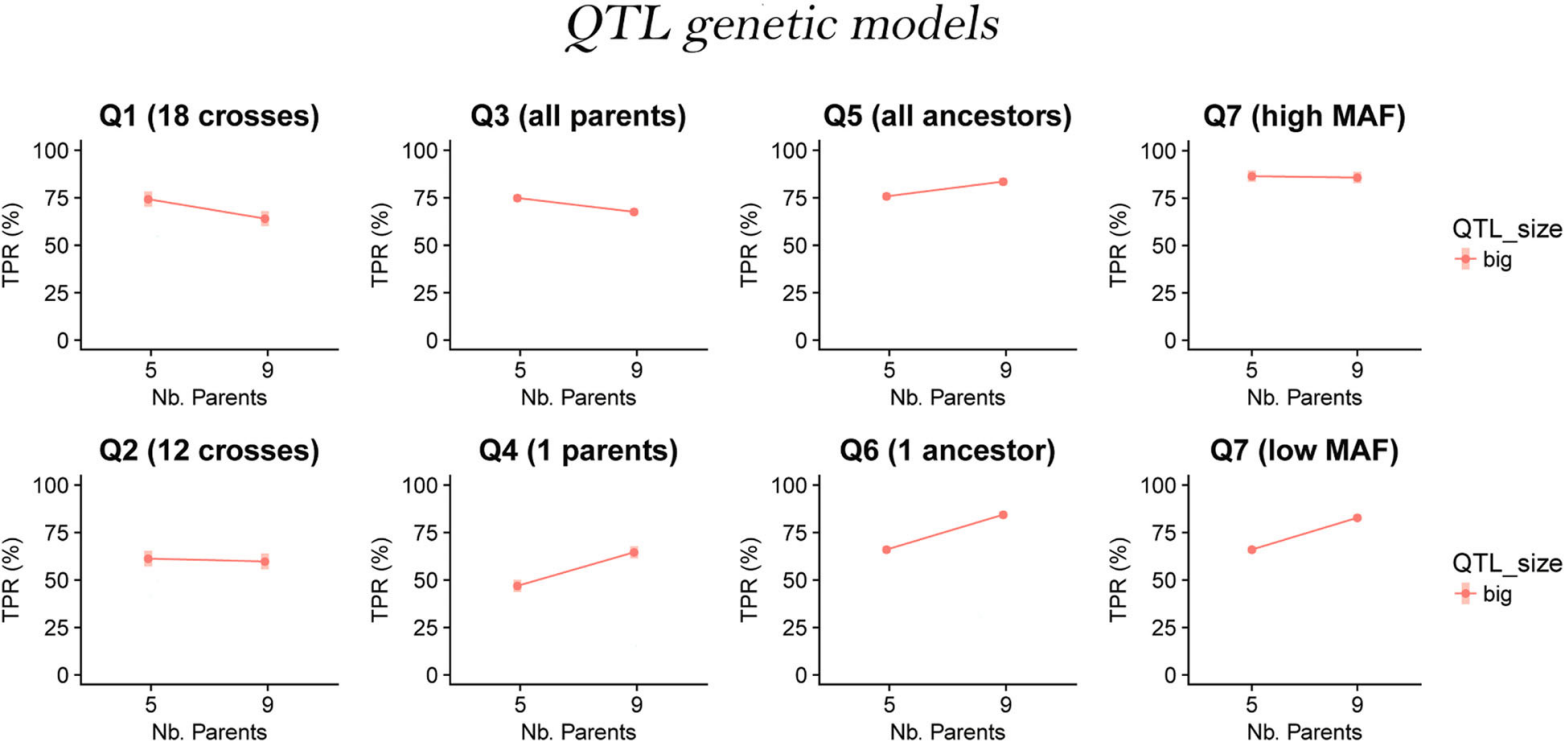
TPR least-squares means over the number of parents (5 and 9) for big (6%) simulated QTLs Q1-7 in populations N = 1600. The bi-allelic QTLs (Q7) were split into a low and a high MAF category given that their MAF was below or above the median

We investigated in more detail the situations where sampling nine parents increased the TPR. In Fig. 5, we plotted the TPR least-squares means against the number of parents for the big (6%) simulated QTLs (Q1 to Q7) in the N = 1600 populations. For the bi-allelic QTLs (Q7), we split the results in a low and a high MAF category given that the QTL MAF was below or above the median of all SNP alleles MAF. For the parental, ancestral and bi-allelic effects we noticed that sampling a larger number of parents was more useful for the QTLs with a reduced number of QTL allelic effects and a reduced MAF (Q4, Q6 and Q7 low MAF). The best example is the difference between Q3 and Q4. Q3 had 9 parental alleles different from zero where Q4 only had one non-zero parental allele. The TPR of Q3 decreased while the TPR of Q4 increased when we sampled nine parents in place of five.

### QTL detection model

**Fig. 6:**
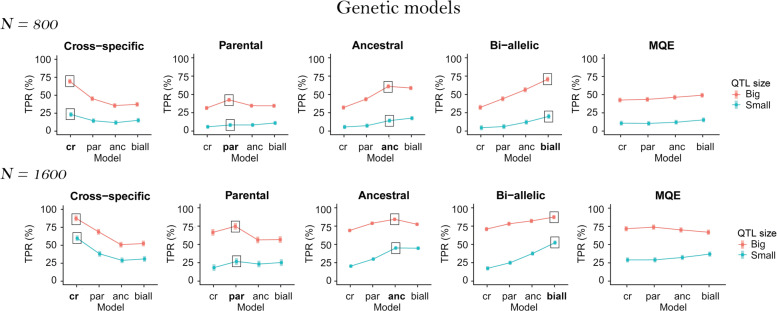
TPR least-squares means over the QTL detection models (cross-specific, parental, ancestral, and bi-allelic) and the QTL sizes (small 2% and big 6%) for all QTL genetic models (cross-specific, parental, ancestral, bi-allelic, and MQE) per population size (N = 800 and N = 1600). The framed results represent the QTL detection model corresponding to the simulated QTL genetic model

In Fig. 6, we plotted the TPR least-squares means from model 1 against the QTL detection model and the QTL sizes. The QTL detection model had a significant effect for all QTL genetic models except for the MQE. The QTL detection model effect was consistent with the way we simulated the QTLs. The QTL detection model that corresponded with the data generating model showed the best performance. For example, ancestral QTLs were detected with the largest TPR using an ancestral model. This result was stronger for the big QTLs.

## Discussion

The main objective of this article was to evaluate the influence of the QTL allelic diversity on MPP QTL detection. We simulated QTLs with different levels of allelic diversity and evaluated the QTL detection power in different MPP designs (diallel, chessboard, factorial, NAM), derived from five or nine parents. Changing the number of parents produced variation in the covered genetic diversity and the number of individuals per cross. We also varied the total population size, the size of the QTL effect, and the QTL detection model used. We evaluated jointly the effect of all parameters in a statistical model to determine their relative contribution, and we determined QTL confidence intervals.

### MPP design

According to the least-squares means (Fig. 3) and the ANOVA results (Additional file 6), the MPP design was mostly important for the cross-specific QTLs. For those QTLs, designs with a reduced number of large crosses like the NAM performed better than designs with many small crosses like the diallel. Obviously, cross-specific QTL effects need large cross sample sizes to be detected. Therefore, to detect QTL effects that are potentially diverse and cross-specific we recommend using designs with few large crosses. Sampling a reduced number of allele genetic background interactions increases the sample size to estimate at least one allelic effect different from zero and therefore detect the QTL.

The MPP design was less important for detecting the other types of QTL allelic effects (parental, ancestral, bi-allelic). Contrary to the cross-specific QTL effects, the parental, ancestral and bi-allelic QTL allelic effects are consistently defined across crosses, which gives them an increased sample size. The parental, ancestral and bi-allelic QTL alleles reached more easily the critical sample size for detection, which made them less dependent on a particular MPP design. This result is consistent with the conclusion of [7] who noticed that the form of the design did not influence the detection of parental and bi-allelic QTLs.

### Number of parents

The number of parents used represents a trade-off between the number of sampled alleles and the sample size to detect the QTLs. For a fixed population size, MPP designs using more parents cover a larger genetic diversity but with a reduced number of individuals per cross. Generally, the TPR decreased with a larger set of parents and reduced cross sizes (Fig. 4). This result was observed for all types of QTL effects in the N=800 populations and for the QTLs with a small effect (2%) in the N=1600 populations. Using MPP designs with large enough crosses is therefore important for QTL detection in MPPs. This result is consistent with several simulations [5, 9, 10, 15] and empirical cross-validation studies [22].

In MPPs composed of crosses, an important part of the QTL variance can happen within crosses rather than between. In our simulation, on average, 54% of the QTL genetic variance happened within crosses (100% for the cross-specific QTLs and around 39% for the other type of QTLs). Moreover, we used a QTL detection model with a cross-specific intercept that accounted for part of the between crosses QTL variance [5, 11]. Therefore, we prefer sampling strategies that increase the sizes of the segregating crosses. In our case, five parents covered probably a sufficient part of the genetic diversity. Thus, resources are better spent on enlarging the sizes of the crosses. This finding is consistent with the conclusions of [7] who showed that QTL detection power reached a plateau after six parents. If few parents already enable to cover a representative sample of the genetic diversity, MPP designs should rather have more individuals per cross than extra crosses (parents).

Still, in some situations, we did find that using nine parents instead of five increased the TPR. This result was consistent with [3, 7, 11, 15]. In our case, we emphasized that using a larger set of parents was only useful to increase the detection power of QTLs with a low MAF (Fig. 5). The parental and ancestral QTLs that only segregated from a single parent (Q4) or ancestral group (Q6) and the low MAF bi-allelic QTLs were the only cases where using nine parents increased the TPR. We hypothesize that when the QTL MAF was low, using more parents enabled to sample at least one cross where the QTL segregated. However, we noticed that the TPR only increased in the large populations (N = 1600) and for the big QTLs. Therefore, the use of more parents should be combined with an increased total population size and will be conditioned by the QTL effect size. A large population and a big QTL effect increase the chance of detection when only few individuals carry the QTL allele.

The results concerning the number of parents illustrate the interest to consider the question of the resource allocation in MPPs given the QTL allelic diversity. In general, we observed that larger crosses increased the TPR for all types of QTL effects in the N=800 populations. The negative effect of cross size reduction was less important for QTLs with shared effects between crosses like the ancestral QTLs. For those QTL alleles, the reduction of the within cross sample size was compensated by an increased between cross sample size. However, in the larger populations (N=1600), we noticed that increasing the number of parents to cover a larger genetic diversity could be useful to detect rare QTLs with a large effect. According to [23], those QTLs effects represent an important part of the genetic variation and are good candidates for marker assisted selection (MAS). Therefore, in recurrent MAS breeding programs with population sizes between 800-1000, we advise to prioritize the enlargement of the cross sizes. However, if the objective is to enrich material with new traits coming from diverse material, it might be worthwhile to increase the covered genetic diversity and the total population size to detect influential alleles.

### Mapping resolution

In Table 3, we noticed that the cross-specific and parental QTLs were subject to larger FDRs and had larger dQTLs than the ancestral and bi-allelic QTLs. To explain those differences, we can emphasize that the cross and parental structures are consistently defined over the offspring genome while the ancestral and the SNP allele distributions vary. Therefore, the correlation between two positions modeled as cross-specific or parental is potentially larger than the one between two ancestral or bi-allelic loci. A reduced correlation between ancestral and bi-allelic loci could explain that less QTL will be wrongly detected away from the true position, which increases the resolution.

The FDR decreased when the detection window size around the simulated QTL increased. For example, around 60% of the QTLs we fail to detect in a 10 cM window around the simulated QTL position are detected if we enlarge the window size to 20 cM. Many false positives could be explained by the extent of the linkage disequilibrium, which is relatively large in *F*_2_ populations. The FDR indicates that, in MPPs composed of *F*_2_ crosses, the confidence interval around a detected QTL should be wide to include the true QTL position. For example, in Table 4, we showed that the 95% empirical confidence interval of the 2% QTLs detected in the N = 800 MQE populations, was equal to 70 cM. For the 6% QTLs the 95% confidence interval was 46 cM. Using a confidence interval of at least 50 cM around the detected QTLs seems to be necessary in MPPs composed of *F*_2_ crosses.

Two reasons can explain the apparent low resolution we obtained. As we can see comparing Table 4 and Additional file 7A, the use of a QTL detection model that does not assume the same type of QTL as the simulated QTL (e.g. use a bi-allelic model to detect cross-specific QTL) increases significantly the CI length, especially for the cross-specific QTLs. This result emphasizes the usefulness of using the correct QTL detection model to improve the resolution and supports the use of strategies aiming at finding the correct dependency structure at the QTL position [17, 24]. We hypothesize that the sample increase from N=800 to N=1800 can increase the number of low-resolution detections of cross-specific QTLs by models with other assumptions. This could explain why for the big cross-specific QTLs the expected increased resolution due to population increase was absent.

The detection of more than one (most of the time two) QTLs per chromosome also has a small impact on the detection resolution (Additional file 7B). In those situations, The QTLs were generally located on both sides of the QTL position with a larger distance to the true position than in the situations where only one QTL is detected. According to our results, the MPPs composed of crosses are not the most desirable with respect to detection resolution. MPPs produced after extra generations of intercrossing, like the MAGIC populations are expected to have a better resolution [14, 25].

### Simulation validity

The low FDR on the chromosome with no simulated QTLs confirms that our QTL detection procedure functioned properly. Extrapolating FDR chr to the whole genome gave us values between 0.9 and 15.3*%* with an average of 5.9*%*. Those results show that a −*log*10(*p*)>4 threshold might be too conservative, especially for a N=800 population. We also compared the TPR we obtained with results from the literature. For example, [26] estimated the QTL detection power of interval mapping method in *F*_2_ populations with 10 QTLs accounting for a total of 30% of the phenotypic variation. This scenario was the closest to our simulation settings. In that case, [26] used a threshold with type I error of 25% and he obtained powers of 57% and 85% for total population sizes of 500 and 1000 individuals, respectively. We compared those values to the TPR obtained with the bi-allelic genetic model scenario detected with the bi-allelic model in the N=800 populations because this scenario was the closest to the one used in [26]. We looked at the TPR with no minimum detection window size around the simulated QTL obtained in the simple interval mapping scan. We used −*log*10(*p*)=3 as significance threshold to account for the larger type I error used by [26]. In that case we obtained a TPR value of 69% comparable to the values obtained by [26] between 57% for N=500 and 69% for N=1000, which supports the credibility of our simulation.

### Application to other types of populations and species

The simulations we performed were based on *F*_2_ crosses starting from real sugar beet data. Our main conclusions were similar to the one of authors that used different types of populations. Indeed, [10] who used *F*_2_, backcross (BC), and full-sibs populations and [15] who used double haploid (DH) reached the same conclusion about the importance of using large crosses. Therefore, we can reasonably consider that our conclusions generalize to other type of populations.

In our simulations, we assumed that all genotypes in a cross came from the same *F*_1_ plant. However, as emphasized by a reviewer, depending on the crop, it might not be possible to generate *F*_2_ crosses containing 450 genotypes from a single *F*_1_ cross. If both parents are inbred, the *F*_1_ will be identical. However, if the parents are partially heterozygous like three of our parents, then *F*_1_ genotypes might differ. If a cross is composed of sub-crosses generated from different homozygous and heterozygous F1 plants, some markers will segregate in parts of the cross (sub-crosses generated from heterozygous F1) and be monomorphic in the rest (sub-crosses generated from homozygous F1). Such a situation is problematic for parental origin determination. In those cases, the best solution is to take into consideration the sub-cross structure for parent origin assignment, which can be done in mppR.

The generalization of our results to other crops would need further confirmations. The use of simulated data starting from real genotypes is a strategy that has been used in several articles [14, 25, 27]. Few of those studies have tested hypotheses about the trade-off between the number of crosses and the number of individuals per cross. Among them, [27] did not find a significant influence of the cross size in simulated MPPs from rapeseed genotypes. The total population size (2000) and the number of simulated QTLs (50) were however different compared to our simulation settings. In [3], the authors simulated 25-100 QTLs on a real maize NAM population with 5000 individuals. They found that increasing the number of parents (crosses) increased the QTL detection power. Assuming that many of the simulated QTLs had a low MAF, this result would be consistent with our second conclusion: increasing the number of parents is beneficial to detect QTLs with low MAF if the total population size is large enough to guarantee a minimum cross size. Since those results represent too few comparison points, we would need other studies to reach firm conclusions about the generalization of our results to other crop species.

## Conclusions

We tried to determine the most powerful design to detect QTLs in MPPs given various levels of QTL allelic diversity. We showed that the trade-off between using a large set of parents to cover a larger genetic diversity with smaller crosses and reducing the number of parents to increase the cross size depends on the type of QTL allelic effects. In most of the cases, sampling a reduced number of parents is enough to cover a sufficient amount of the genetic diversity. Therefore, resources should be used to increase cross sizes rather than increasing the covered allelic diversity. However, when the main goal is the detection of QTLs with rare alleles and large phenotypic effects, using a larger set of parents can be beneficial given that the total population size is also increased. Concerning the QTL detection resolution in MPPs composed of *F*_2_ crosses, we noticed that those populations have a low resolution. We advise to use a confidence interval of at least 50 cM around the detected QTL positions to have a reasonable probability that it includes the true QTL position.

## Supplementary Information

**Additional file 1** Figure genetic map information. Genetic map plot with marker density information (PDF 140 kb).

**Additional file 2** Figure simulated QTLs. Visualisation of the different simulated QTL effects (PDF 2833 kb).

**Additional file 3** Text file phenotype simulation procedure. Detailed description of the procedure used to simulate the phenotypic values (PDF 112 kb).

**Additional file 4** R script simulation reproduction. R script allowing to reproduce the simulation used in the article (R 10 kb).

**Additional file 5** Histograms distance to the QTL. Histograms of the distribution values of the distance between the simulated and detected QTL (PDF 46 kb).

**Additional file 6** ANOVA results. ANOVA F-statistics describing the effect of the MPP design, the number of parents, the QTL detection model, the QTL size, and the QTL effect on the TPR at 10 cM for the N = 800 and N = 1600 populations (PDF 73 kb).

**Additional file 7** Empirical confidence intervals. A) Empirical QTL detection confidence intervals using only the results when generating and detection QTL model are the same. B) Confidence intervals for chromosomes with only one detected QTL (PDF 37 kb).

## Data Availability

The datasets generated and analysed during the current study are not publicly available due to KWS SAAT SE restrictions. However, a script allowing the reproduction of the simulation using genetic data from the EU-NAM population is available in Additional file 4. The datasets as well as the R package and script are available in the following figshare repository [https://figshare.com/articles/dataset/Supplementary_material_from_The_influence_of_QTL_allelic_diversity_on_QTL_detection_in_multi-parent_populations_a_simulation_study_in_sugar_beet_/13370549]. The R package and the data are also available in Vincent Garin github repository [https://github.com/vincentgarin/mppSim].
