# Supplementary material for: The influence of QTL allelic diversity on QTL detection in multi-parent populations: a simulation study in sugar beet
Source: BMC Genom Data. 2021 Feb 3;22:4. doi: 10.1186/s12863-021-00960-9 (PMC7860181; doi:10.1186/s12863-021-00960-9)
Supplement: Supplementary file 1 — Additional file 1 Figure genetic map information. Genetic map plot with marker density information (PDF 140 kb). [file 12863_2021_960_MOESM1_ESM.pdf]

Genetic map used to simulate the QTL effects

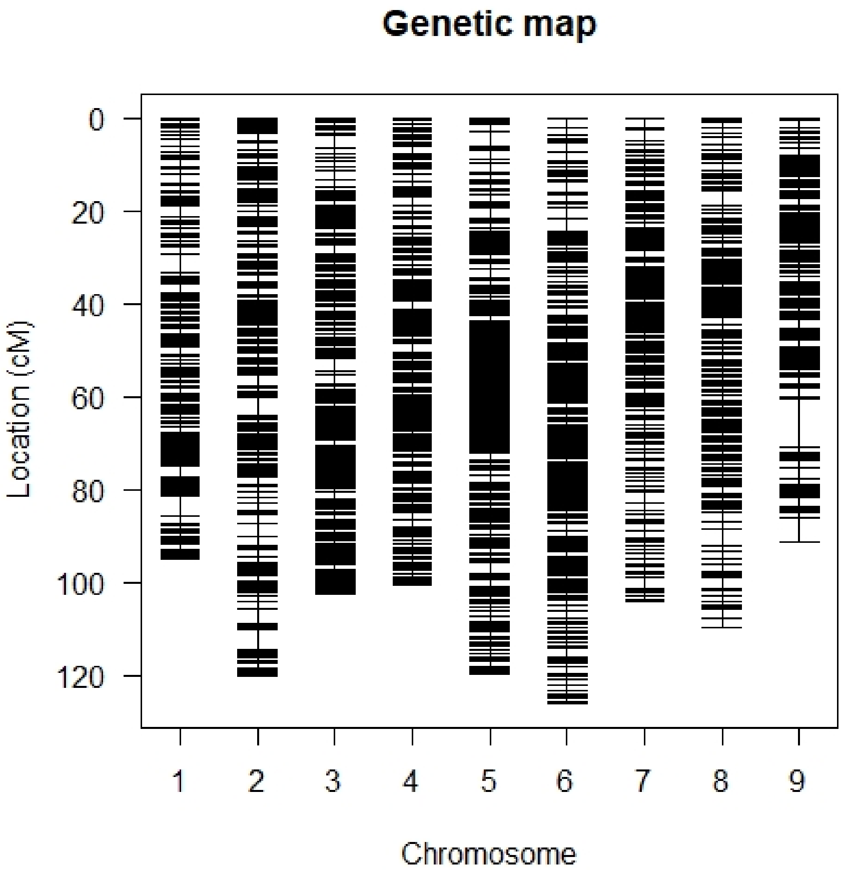

Chromosome	N	Length(cM)	Average spacing	maximum spacing
1	424	94.7	0.2	4.3
2	390	120.2	0.3	4.2
3	694	102.5	0.1	3.0
4	577	100.5	0.2	2.2
5	676	119.5	0.2	3.3
6	705	126.0	0.2	3.0
7	585	103.9	0.2	2.4
8	509	109.7	0.2	3.5
9	440	91.3	0.2	10.6
Overal	5000	968.3	0.2	10.6
