# Supplementary figures and images for: The influence of QTL allelic diversity on QTL detection in multi-parent populations: a simulation study in sugar beet
Source: BMC Genom Data. 2021 Feb 3;22:4. doi: 10.1186/s12863-021-00960-9 (PMC7860181; doi:10.1186/s12863-021-00960-9)

# Illustration of the simulated QTL effect on the reference diallel

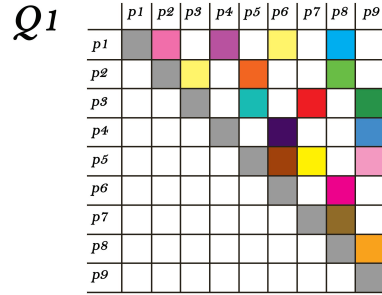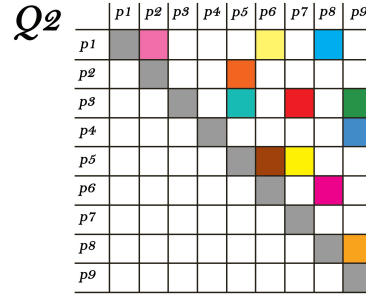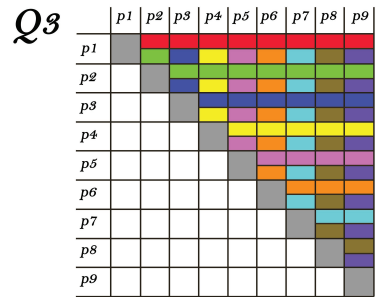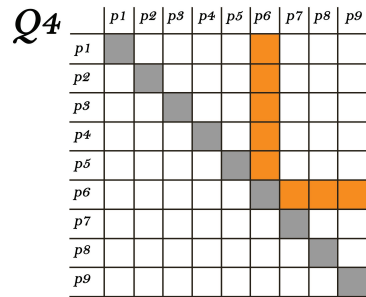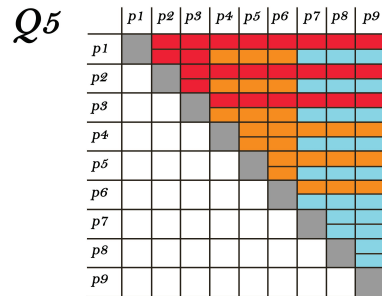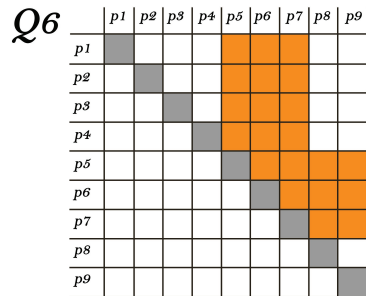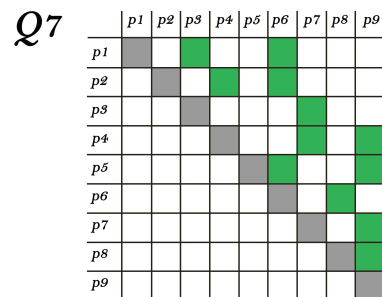

Supplement: Supplementary file 2 — Additional file 2 Figure simulated QTLs. Visualisation of the different simulated QTL effects (PDF 2833 kb). [file 12863_2021_960_MOESM2_ESM.pdf]
