# Supplementary material for: The influence of QTL allelic diversity on QTL detection in multi-parent populations: a simulation study in sugar beet
Source: BMC Genom Data. 2021 Feb 3;22:4. doi: 10.1186/s12863-021-00960-9 (PMC7860181; doi:10.1186/s12863-021-00960-9)
Supplement: Supplementary file 3 — Additional file 3 Text file phenotype simulation procedure. Detailed description of the procedure used to simulate the phenotypic values (PDF 112 kb). [file 12863_2021_960_MOESM3_ESM.pdf]

Phenotypic values simulation procedure

For each realizations, phenotypic values were simulated at the level of the reference diallel population (36 crosses with 450 genotypes). The ith realization included the following steps:

1. The phenotypic variance was expressed in terms of QTL and random error variance $\sigma_p^2 = \sigma_Q^2 + \sigma_e^2$. We assumed a strict additivity of these components.

2. We randomly sampled the 8 QTL positions $(q_1, ..., q_8)$. Each QTL was on a different chromosome. We assumed that the QTL positions were independent and that the global QTL variance (σ_Q^2) was the sum of each individual QTL variance contribution $(\sigma_Q^2 = \sum_{i=1}^{n_{QTL}} \sigma_{qi}^2)$. We calculated the individual QTL variance using $\sigma_{qi}^2 = V(\boldsymbol{X_{q_i}\beta_i})$ where X_{q_i} and $\boldsymbol{\beta_i}$ are the incidence matrix and the allelic effect of QTL i. The incidence matrix X_{q_i} took different forms according to the type of simulated QTL effect (cross-specific, parental, ancestral, bi-allelic). The form of $\boldsymbol{\beta_i}$ followed the definition of the simulated QTLs (Q1-7). The non-zero elements of $\boldsymbol{\beta_i}$ were sampled from a uniform distribution (1-10) with random sign assignment. We scaled the $\boldsymbol{\beta_i}$ values to make sure that the σ_{qi}^2 reached the desired phenotypic proportion (2 or 6 %). Finally we calculated the QTL contribution to the phenotype using $\boldsymbol{y_Q} = \boldsymbol{X_Q\beta}$.

3. We determined the error variance contribution (σ_e^2) such that $\sigma_e^2 = ((1 - h^2)/h^2) * \sigma_Q^2$. In all cases, h^2 was equal to 0.32. Given σ_e^2 we sample the phenotypic variation due to the error using $\boldsymbol{y_e} \sim N(0, \sigma_e^2)$. The simulated phenotypic values were therefore expressed as $\boldsymbol{y_{sim}} = \boldsymbol{y_Q} + \boldsymbol{y_e}$.

4. In many cases, even if the QTLs were sampled on different chromosomes, there was still an important covariance between the QTL positions. Therefore, we sampled a large number of realizations and we kept only the one where the covariance between the QTL positions was inferior to 1% of the total phenotypic variance.
