# Supplementary material for: The influence of QTL allelic diversity on QTL detection in multi-parent populations: a simulation study in sugar beet
Source: BMC Genom Data. 2021 Feb 3;22:4. doi: 10.1186/s12863-021-00960-9 (PMC7860181; doi:10.1186/s12863-021-00960-9)

Histograms of the distance between simulated and detected QTL distributions

The red, blue, and black dashed lines represent the 90, 95 and 99 distribution percentiles.

N = 800

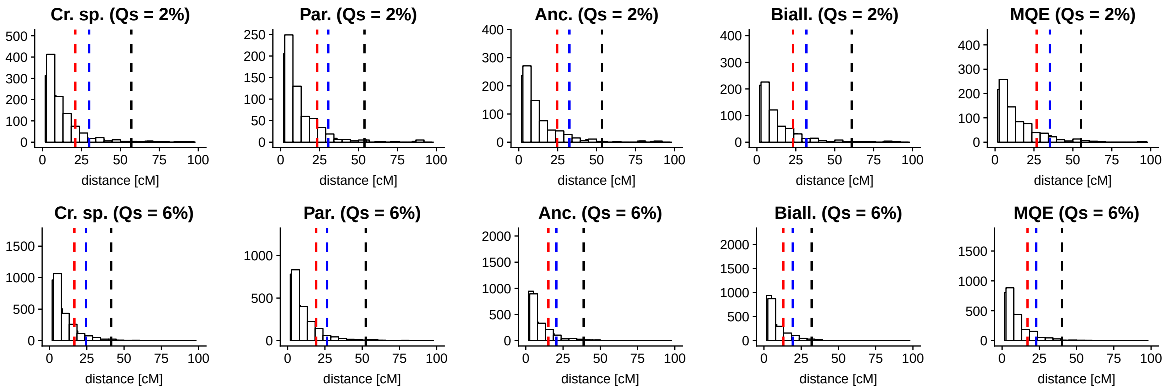

N = 1600

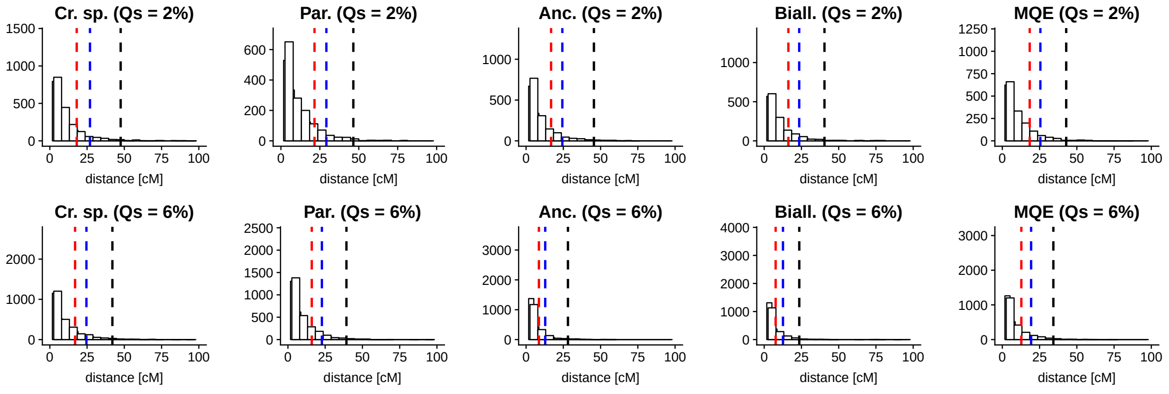

Supplement: Supplementary file 5 — Additional file 5 Histograms distance to the QTL. Histograms of the distribution values of the distance between the simulated and detected QTL (PDF 46 kb). [file 12863_2021_960_MOESM5_ESM.pdf]
