# Supplementary material for: The influence of QTL allelic diversity on QTL detection in multi-parent populations: a simulation study in sugar beet
Source: BMC Genom Data. 2021 Feb 3;22:4. doi: 10.1186/s12863-021-00960-9 (PMC7860181; doi:10.1186/s12863-021-00960-9)
Supplement: Supplementary file 6 — Additional file 6 ANOVA results. ANOVA F-statistics describing the effect of the MPP design, the number of parents, the QTL detection model, the QTL size, and the QTL effect on the TPR at 10 cM for the N = 800 and N = 1600 populations (PDF 73 kb). [file 12863_2021_960_MOESM6_ESM.pdf]

A. ANOVA results N=800 populations

Table 1: ANOVA degrees of freedom (df), and F-statistics with significance of the MPP design, the number of parents, the QTL detection model, the QTL size and the QTL effect on TPR per QTL genetic model (cross-specific, parental, ancestral, bi-allelic, and MQE) for the N = 800 populations. Adjusted and cross-validation R2 of the models.

	df	Cr. sp.		Parental		Ancestral		Bi-allelic		MQE	
MPP des	3	91.9	***	2.6		1.4		3.4	*	15.6	***
Nb. par	1	188.6	***	131.8	***	37	***	22.4	***	97	***
Det. model	3	75.5	***	8.9	***	87.4	***	99	***	5	**
QTL size	1	693.3	***	845.1	***	1372.9	***	1167.8	***	824	***
QTL type	$0 - 3^a$	8.5	**	0		0				10.9	***
MPP des x Nb. par	3	9.7	***	1.8		1.3		0.1		3.4	*
MPP des x Det. model	9	4.5	***	1.2		1.9		1.5		0.5	
MPP des x QTL size	3	2.4		3.1	*	11.2	***	0.8		0.3	
Nb. par x Det. model	3	3.2	*	2.5		13.9	***	13.3	***	2.3	
Nb. par x QTL size	1	5.3	*	22	***	1.1		1.4		3.5	
Det. model x QTL size	3	22	***	6.3	***	17.8	***	17.3	***	0.2	
Residuals	$94 - 97^a$										
adj. R2 (%)		92		89		93		96		67	
CV R2 (%)		90		85		91		92		65	

* p < 0.05; ** p < 0.01; *** p < 0.001

a. The df of the QTL type term is equal to 1, 1, 1, 0, 3 from the Cr. sp. to the MQE genetic model. The df of the residuals change according to the df of the QTL type term.

B. ANOVA results N=1600 populations

Table 2: ANOVA degrees of freedom (df), and F-statistics with significance of the MPP design, the number of parents, the QTL detection model, the QTL size and the QTL effect on TPR per QTL genetic model (cross-specific, parental, ancestral, bi-allelic, and MQE) for the N = 1600 populations. Adjusted and cross-validation R2 of the models.

	df	Cr. sp.		Parental		Ancestral		Bi-allelic		MQE	
MPP des	3	44.8	***	1		4.4	**	0.8		4.4	**
Nb. par	1	69.6	***	4.5	*	0		1.6		13.2	***
Det. model	3	122.2	***	9.6	***	113	***	125	***	0.3	
QTL size	1	328.3	***	623.8	***	2614.8	***	2217.6	***	952.7	***
QTL type	$0 - 3^a$	28.2	***	17.1	***	13	***			10.9	***
MPP des x Nb. par	3	19.9	***	0.5		4.5	**	4	*	5.9	***
MPP des x Det. model	9	5.9	***	1		5.5	***	3.5	**	0.3	
MPP des x QTL size	3	4.9	**	0.1		0.4		0.5		1.9	
Nb. par x Det. model	3	8.9	***	1		17.6	***	16.5	***	1.3	
Nb. par x QTL size	1	17.3	***	28.7	***	242.9	***	54.9	***	44.7	***
Det. model x QTL size	3	2.6		8.2	***	22.7	***	20	***	6.8	***
Residuals	$94 - 97^a$										
adj. R2 (%)		89		85		96		98		69	
CV R2 (%)		85		80		95		95		66	

* p < 0.05; ** p < 0.01; *** p < 0.001

a. The df of the QTL type term is equal to 1, 1, 1, 0, 3 from the Cr. sp. to the MQE genetic model. The df of the residuals change according to the df of the QTL type term.
