# Supplementary material for: The influence of QTL allelic diversity on QTL detection in multi-parent populations: a simulation study in sugar beet
Source: BMC Genom Data. 2021 Feb 3;22:4. doi: 10.1186/s12863-021-00960-9 (PMC7860181; doi:10.1186/s12863-021-00960-9)
Supplement: Supplementary file 7 — Additional file 7 Empirical confidence intervals. A) Empirical QTL detection confidence intervals using only the results when generating and detection QTL model are the same. B) Confidence intervals for chromosomes with only one detected QTL (PDF 37 kb). [file 12863_2021_960_MOESM7_ESM.pdf]

A. Confidence intervals when generating and detection QTL model are the same

Table 1: Empirical confidence intervals in cM based on the 90, 95 and 99 percentile values from the dQTL distribution per QTL size (small 2% and big 6%), per QTL genetic model (cross-specific, parental, ancestral, bi-allelic, and MQE), and per population size (N = 800 and N = 1600).

		small QTLs (2%)				big QTLs (6%)			
		Cr. sp.	Par.	Anc.	Biall.	Cr. sp.	Par.	Anc.	Biall.
	.90	33	38	49	42	21	28	24	19
N=800	.95	48	49	63	59	30	40	36	34
	.99	98	62	90	110	49	77	69	62
	N det. QTL	480	179	325	429	1236	828	1114	1243
	% det. QTL	30	11	20	27	77	52	70	78
	.90	21	36	29	26	11	19	12	8
N=1600	.95	31	49	44	41	17	26	19	14
	.99	69	81	81	75	35	45	37	37
	N det. QTL	1072	551	863	975	1451	1310	1412	1442
	% det. QTL	67	34	54	61	91	82	88	90

B. Confidence intervals for chromosomes with only one detected QTL

Table 2: Empirical confidence intervals in cM based on the 90, 95 and 99 percentile values from the dQTL distribution per QTL size (small 2% and big 6%), per QTL genetic model (cross-specific, parental, ancestral, bi-allelic, and MQE), and per population size (N = 800 and N = 1600).

		small QTLs (2%)					big QTLs (6%)				
		Cr. sp.	Par.	Anc.	Biall.	MQE	Cr. sp.	Par.	Anc.	Biall.	MQE
	.90	42	47	47	45	49	32	37	30	26	32
N=800	.95	57	61	62	63	68	45	52	40	38	43
	.99	106	108	105	136	110	79	98	74	63	75
	N det. QTL	1369	773	1000	924	1094	3535	2955	3635	3692	3468
	% det. QTL	21	12	16	14	17	55	46	57	58	54
	.90	35	42	31	31	34	31	31	16	15	23
N=1600	.95	48	55	45	46	48	45	43	24	24	36
	.99	86	89	84	75	77	77	76	54	47	64
	N det. QTL	3015	2026	2674	2541	2510	4566	4799	5216	5349	4939
	% det. QTL	47	32	42	40	39	71	75	82	84	77
